# Vessel co-option in glioblastoma: emerging insights and opportunities

**DOI:** 10.1007/s10456-019-09691-z

**Published:** 2019-11-02

**Authors:** Giorgio Seano, Rakesh K. Jain

**Affiliations:** 1 Tumor Microenvironment Laboratory, Institut Curie Research Center, Paris Saclay University, PSL Research University, Inserm U1021, CNRS UMR3347, Orsay, 91405 France; 2 Edwin L. Steele Laboratory, Department of Radiation Oncology, Massachusetts General Hospital and Harvard Medical School, Boston, MA 02114 USA

**Keywords:** Vessel co-option, Glioblastoma, Anti-angiogenesis, Cell migration, Tumor microenvironment

## Abstract

Vessel co-option is the movement of cancer cells towards and along the pre-existing vasculature and is an alternative to angiogenesis to gain access to nutrients. Vessel co-option has been shown as a strategy employed by some glioblastoma (GBM) cells to invade further into the brain, leading to one of the greatest challenges in treating GBM. In GBM, vessel co-option may be an intrinsic feature or an acquired mechanism of resistance to anti-angiogenic treatment. Here, we describe the histological features and the dynamics visualized through intravital microscopy of vessel co-option in GBM, as well as the molecular players discovered until now. We also highlight key unanswered questions, as answering these is critical to improve understanding of GBM progression and for developing more effective approaches for GBM treatment.

## Introduction

Tumors depend on blood vessels for growth and dissemination, making the tumor vasculature a compelling therapeutic target to limit tumor growth and metastasis. Angiogenesis, which is the formation of new vessels from pre-existing ones, is the most well-studied mechanism for tumors to generate vasculature. However, some tumors grow in already highly vascularized organs like liver, lung, lymph nodes, and brain, reducing the need to induce angiogenesis. As a consequence, tumor cells that are infiltrative have easy access to well-perfused pre-existing blood vessels. Vessel co-option, which we define here as the movement of tumor cells towards and then along the pre-existing blood vessels, is one mechanism to access the vasculature.

A compelling example of an infiltrating tumor is glioma—a highly vascularized tumor. Gliomas are the most common malignant primary tumors growing in the central nervous system in adults, constituting approximately 80% of the malignant cases [1]. Gliomas can be divided into three sub-classes: oligodendrogliomas, astrocytomas, and glioblastomas (GBMs). GBM is one of the most deadly types of cancer with a median overall survival of 15 months [2, 3]. Despite massive clinical and research efforts, GBM treatment remains one of the most challenging tasks in clinical oncology [4]. Since the brain tissue is highly vascularized, just 3–6 glioma cells are needed to fill the space between two adjacent microvessels [5]. This estimation stresses the ease with which GBM cells could be in contact with already well-perfused blood vessels without the need to activate the pathways of tumor angiogenesis. Vessel co-opting GBM cells benefit from both their oxygen and nutrient supply and the specific vascular niche microenvironment that stimulates proliferation and self-renewal mediated by crosstalk with the cellular components of blood vessels. Moreover, infiltrating GBM cells employing vessel co-option use the vasculature as a scaffold to invade into normal CNS tissue.

There are a number of preclinical studies showing that several other types of tumor cells may migrate along the normal pre-existing vasculature [6–8], such as non-small-cell lung cancer [9] and uveal melanoma [10, 11], as well as metastases from melanoma, breast, and colorectal cancer in the lymph node [12, 13], liver [14–16], or brain [15, 17–19]. Thus, vessel co-option may be a widespread and general strategy of growth for infiltrating tumors.

## Vessel co-option as one of the GBM spreading strategies

**Fig. 1:**
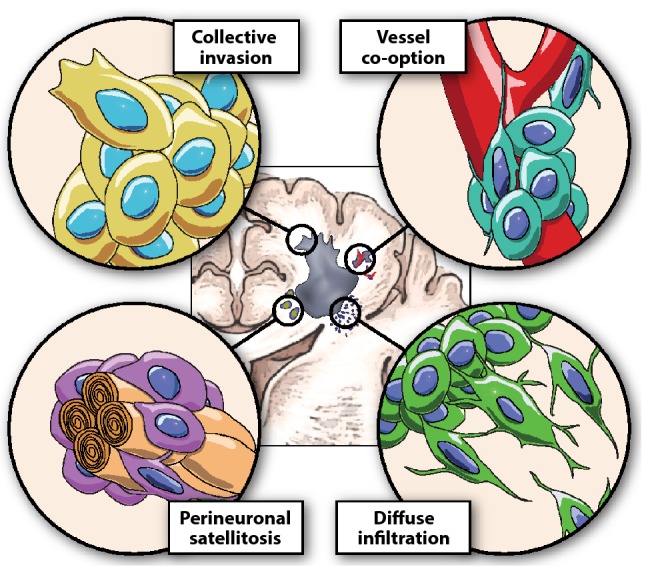
Strategies used by GBM for infiltration into the surrounding brain tissue. Collective invasion of the surrounding tissue, perivascular migration (also called as vessel co-option), peri-neuronal satellitosis, and individual-cell migration within the extracellular matrix (diffuse infiltration)

Almost all GBM patients experience tumor recurrence, typically occurring within 1–2 cm of the original tumor border [20]. Indeed, non-proliferating, infiltrating GBM cells [21] move very fast in the surrounding tissue with a velocity of 2 to 6 μm/h in preclinical models [22]. Their migration is characterized by a diffuse, fast, and undirected movement or by a slower, invasive, directional migration [22]. Histologically, GBM cells infiltrate the surrounding tissue through four different invasion pathways: (i) individual-cell migration within the extracellular matrix (diffuse infiltration), (ii) collective invasion of the surrounding tissue, (iii) peri-neuronal satellitosis, and (iv) perivascular migration (also called as vessel co-option) [8, 22–24] (Fig. 1). Specifically, vessel co-option has been hypothesized to be a significant contributor to recurrence and lethality as it enables the formation of microscopic tumor extensions beyond the margins of surgical resection [25, 26]. Tumor infiltration is probably one of the most clinically relevant biological consequences of vessel co-option in GBMs. Indeed, vessel co-option has been shown as a preferred invasion strategy for some GBM cell lines [5]. However, the studies performed until now are limited as they comprise few patient-derived or syngeneic cell lines [e.g., C6 (rat), GL261 (mouse), and D54 and MGG8 (patient derived)], and thus, are unable to reflect the high complexity of GBM where different subtypes are present with an evident inter- and intra-tumor heterogeneity [27]. It is thus conceivable that different subtypes could employ distinct invasion strategies resulting in a more complex scenario where in the same tumor we encounter areas, subpopulations, or individual cells that differentially prefer vessel co-option, peri-neuronal satellitosis, individual infiltration, or collective invasion.

## Insights from in vivo imaging of vessel co-option

Initially, the existence of vessel co-option was deduced from examination of histological tissue sections [28, 29]. Indeed, (i) the frequent vascular association of infiltrating tumor cells, as occurring in perivascular satellitosis, (ii) the presence of normal blood vessels and (iii) the maintenance of the normal vascular architecture in the infiltrated areas are signs of bona fide vessel co-option in GBMs; but they do not reveal the dynamics of the movement of tumor cells towards pre-existing vasculature. In the last decade, improvements in imaging technologies have made it possible to visualize vessel co-option in organotypic cultures and in live animals.

### Organotypic brain slices

In order to observe the ex vivo dynamics, GBM vessel co-option has been recapitulated using organotypic brain slices cultured with patient-derived GBM cells [30]. Interestingly, GBM cells get in contact with blood vessels in this setup and this model shows interesting features of vessel co-option. First, GBM cells are “attracted” by blood vessels even without a gradient of nutrients and oxygen, since blood vessels are not perfused in brain slices. This suggests that there are chemoattractants produced and released by blood vessels, as discussed below. Secondly, the interaction between tumor cells with components of blood vessels is very dynamic. Moreover, one study revealed that GBM cells employ Cdc42-dependent and actin-based cytoplasmic extensions to modify the normal activity of pericytes around capillaries [30].

### Imaging of serially excised tissue

Another interesting report sheds light on the in vivo occurrence of vessel co-option. The authors quantified the GBM cells’ association with blood vessels by analyzing the histology of multiple animals sacrificed at different intervals from tumor implantation [5]. The syngeneic GL261 GBM model at the early stage of tumor progression showed massive perivascular invasion in the infiltrative area [5].

An additional study analyzed multiple patient-derived cell lines implanted in nude mice and demonstrated that GBM cells at the infiltrative area are associated more with capillaries (less than 7-μm-diameters) than venules/arterioles [31]. Moreover, ultra-structural micrographs of GBM cells associated with blood vessels showed clear cell-to-cell contacts of GBM cells with endothelial cells [31].

### In vivo imaging

**Fig. 2:**
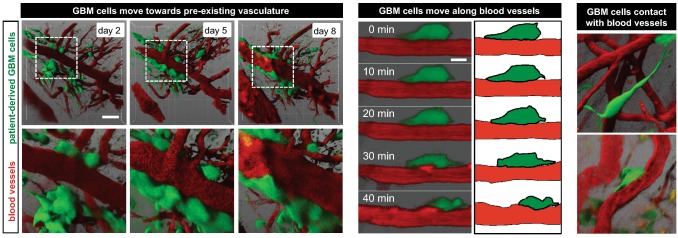
Intravital microscopy of GBM vessel co-option dynamics. Time-lapse imaging demonstrating that GBM cells move towards and then along the pre-existing blood vessels, maintaining very close contact. Reproduced and adapted from [25]

The intravital microscopy can provide powerful insights into the dynamics of vessel co-option. Two studies using intravital microscopy—one from our laboratory and the other from Dr. Frank Winkler’s Laboratory—showed that GBM cells preferentially use blood vessels in the GL261 mouse glioma model to spread in a directional manner [32, 33]. Vessel co-opting GBM cells were frequently found next to multiple capillary structures where microvessels are parallel [32] and were strongly increased by anti-angiogenesis treatment [33]. Our second intravital time-lapse imaging study of patient-derived GBM cells implanted orthotopically showed that GBM cells closely interact with blood vessels, and move towards and then along the pre-existing brain vasculature [25] (Fig. 2). Vessel co-option appears to occur predominantly at the tumor–brain interface and in some GBM subtypes it is a fairly frequent phenomenon occurring in more than 50% of cells. Intravitally, as previously shown on brain slices, GBM cells form close and dynamic contacts with GFP-tagged endothelium [25].

## Vessel co-option molecular pathways

Although the existence of vessel co-option has been hypothesized for some time [28, 29], the molecular pathways involved in the process of GBM vessel co-option are beginning to emerge now (Table 1).

**Table 1:** Vessel co-option pathways in GBMs

Pathways	Experimental model	Notes	References
Bradykinin	Patient-derived D54 in vivo model and in vitro co-culture	Bradykinin is released from blood vessels, while GBM cells express B2R, their inhibition impairs vessel co-option	[34, 35]
CXCR4/SDF1α	Gl261 mouse in vivo model and in vitro co-culture	SDF1α is expressed in neuronal and endothelial cells, while GBM cells express CXCR4, their inhibition impairs vessel co-option and radiosensitizes tumors	[33, 37–39]
Ang-2	C6 rat in vivo model	Ang-2 and VEGF are expressed in vessel co-option areas as a consequence of vascular regression	[33, 43]
IL-8	In vitro co-culture and in vivo implants	Endothelial cells increase GBM invasiveness and tumor growth through IL-8-mediated enrichment of glioma stem cells	[46, 47]
EGFRvIII	Mouse in vivo model and ex vivo brain slice	EGFRvIII-hi GBM cells are highly vessel co-opting and tumors originated by them are highly infiltrative and aggressive	[49]
MDGI/FABP3	Mouse in vivo model and ex vivo brain slice	Modulation of MDGI/FABP3 strongly alters the GBM cells’ ability of infiltrating the surrounding brain tissue with perivascular migration	[51, 52]
IRE-1α	Neurospheres and U87 in vivo models	Inhibition of IRE-1α increases vessel co-option and decreases pro-angiogenic pathways	[54, 55]
CDC42	Ex vivo brain slice	Vessel co-opting GBM cells co-opt and interact with pericytes in a CDC42-dependent manner	[30]
EphrinB2	Patient-derived MGG8 in vivo model, syngeneic model and in vitro co-culture	Endothelial Ephrin-B2 regulates vessel co-option, when, and only if, the ligand Ephrin-B2 is upregulated in GBM cells	[58]
Olig2/Wnt7a	Patient-derived MGG8 in vivo model, EGFRvIII-induced syngeneic model and ex vivo brain slice	Olig2-Wnt7 axis drives individual vessel co-option in oligodendrocyte-like GBM cells, its inhibition impairs vessel co-option and chemosensitizes tumors	[18, 25]

*CXCR4* CXC receptor-4, *SDF1α* stromal cell-derived factor-1α, *IL-8* interleukin 8, *Ang-2* angiopoietin 2, *CDC42* cell division control protein 42, *EGFRvIII* epidermal growth factor receptor variant III, *MDGI*/*FABP3* mammary-derived growth inhibitor (MDGI)/fatty acid binding protein 3, *IRE1α* inositol-requiring enzyme (IRE)-1α, *Wnt* is acronym of homologous wingless (wg) and Int-1, *Olig2* oligodendrocyte transcription factor

### Bradykinin

Bradykinin, a member of the kinins, is an endothelial cell-cleaved product of high molecular weight kininogen [34]. Bradykinin is physiologically present in the brain and is increased during tumor progression. Moreover, vessel co-opting GBM cells expresses high levels of bradykinin receptor-2 (B2R) [34]. Bradykinin induces chemotaxis in GBM cells and increases the GBM invasion of the surrounding brain tissue [35]. Interestingly, pharmacological or genetic inhibition of B2R in GBM cells impairs vessel co-option [34]. Of note, the bradykinin receptor inhibitor Icatibant, already FDA-approved for the treatment of acute attacks of hereditary angioedema, may be an interesting option for novel anti-vessel co-option treatment in GBM [23].

### CXCR4/SDF-1α

Stromal cell-derived factor (SDF)-1α, also known as CXCL12, is a member of the CXC subfamily of chemokines and interacts with the seven-transmembrane G-protein-coupled receptor CXCR4. Originally, chemokines and their receptors were shown to be potent regulators of chemotaxis and trans-endothelial migration in leukocytes. They have also been described as potential chemotactic cues in tumors [36]. SDF1α has been shown to be expressed in neurons, blood vessels, and white matter tracks, and all components of the GBM secondary events of satellitosis. Moreover, stimulation with vascular endothelial growth factor (VEGF), typically present at the tumor–brain interface, upregulates SDF1α in neurons and endothelial cells, while CXCR4 was found to be overexpressed in invading GBM cells [37]. In vitro, CXCR4+ GBM cells migrate towards a gradient of SDF1α and inhibition of CXCR4 reduces GBM migration. Genetic or pharmacological inhibition of CXCR4 reduces invasion and improves survival in GBM as well as radiosensitizes tumors as measured by mouse survival and cell apoptosis [38]. Importantly, the SDF1α/CXCR4 pathway was found to be upregulated by anti-angiogenic treatment [39], suggesting reciprocity between angiogenesis and vessel co-option (see below).

### Angiopoietin-2

Angiopoietin-2 (Ang-2) and VEGF are the most important pro-angiogenic factors, produced and released by many tumors as a consequence of hypoxia in order to stimulate the formation of new blood vessels [40–42]. Although apparently counterintuitive, these mainly pro-angiogenic pathways have been shown to be present in vessel co-option areas at early stages of GBM formation. Indeed, Ang-2 is highly expressed in co-opted blood vessels in the C6 rat glioma model [43]. This report also described that after co-option multiple blood vessels regress with a consequent avascular tumor stage. During this vascular regression, the co-opting GBM cells begin expressing high levels of VEGF [43] and this may be the consequence of a reduction of perfusion in the co-opted/regressing blood vessels, with resulting hypoxia. Using a mathematical model, we described the dynamics of vessel co-option and showed that the vessel regression is caused by compression of vessels by the growth of cancer cells around co-opted vessels [33]. Although multiple other studies reported that vessel co-option is independent of anti-VEGF treatment or even induced by it [5, 14, 15, 25, 44], the precise temporal role of Ang-2 and VEGF in vessel co-option areas and the dynamics of vascular regression of co-opted blood vessels needs to be further investigated using intravital microscopy.

### Interleukin-8

Interleukin-8 (IL-8) is a pro-inflammatory chemokine important in the initiation of neutrophil chemotaxis and degranulation. The receptors for it are two cell-surface G-protein-coupled receptors (CXCR1 and CXCR2). IL-8 has been shown to be particularly important for tumor progression and upregulates stem cell marker expression in GBM and other cancers [45]. In two distinct reports, co-culture of patient-derived GBM and endothelial cells was used to discover the chemotactic pathways activated by endothelial cells to stimulate GBM cell invasion. The authors showed that endothelial IL-8 increased GSCs invasiveness and growth [46, 47]. Moreover, another report demonstrated that the IL-8-CXCR1/2 axis induces GBM proliferation, invasion, and vascular mimicry [48].

### EGFRvIII

EGFRvIII, a mutation isoform formed by the deletion of exons 2-7 of the epidermal growth factor receptor (EGFR), is a common alteration in GBM. Using brain slice and in vivo orthotopic models, GBM cells with high EGFRvIII expression have been shown to be highly vessel co-opting and the tumors originated from them are highly infiltrative and aggressive [49]. This report did not investigate the molecular mechanisms of action of EGFRvIII-mediated vessel co-option, but suggests potential involvement of migration pathways and reduction of ECM adhesion [49].

### MDGI/FABP3

Mammary-derived growth inhibitor (MDGI), also called heart-type fatty acid binding protein (H-FABP/FABP3), enables the intracellular transport of fatty acids [50]. MDGI/FABP3 was found overexpressed in aggressive mesenchymal GBM and the tumor vasculature, which correlated with poor patient survival [51, 52]. Notably, modulation of MDGI/FABP3 strongly altered the GBM cells’ ability to co-opt blood vessels as evident in the histological analysis [52]. Pharmacological targeting of the MDGI/FABP3 pathway using the antihistamine Clemastine strongly inhibited perivascular migration and invasive growth [52].

### Inositol-requiring enzyme (IRE)-1α

Inositol-requiring enzyme (IRE)-1α is an endoplasmic reticulum transmembrane protein and a cellular stress sensor [53]. Selective inactivation of IRE-1α RNAse in GBM cells using dominant-negative approaches induces vessel co-option in the U87 tumor model and increases in vitro migration [54]. Interestingly, inhibition of IRE1α also decreases all pathways for angiogenesis [55], thus confirming the hypothesis of angiogenesis/co-option reciprocity (see below).

### CDC42

CDC42 is a key molecular actor for directional migration and drives the formation of filopodia at the leading edge of cells [56]. Using brain slices co-cultured with GBM cells, a report showed a close CDC42-dependent interaction between GBM cells and pericytes in co-opted vessels [30]. The report further showed that targeting Cdc42 function impairs GBM vessel co-option [30]. Although an interesting finding, CDC42 inhibition modifies the migratory and contractive ability of the GBM cells, inhibiting all migratory/infiltration strategies and not specifically vessel co-option.

### Ephrin-B2

Ephrin-B2 is a member of the Eph/ephrin family, a fundamental cell-to-cell communication system with widespread roles in tissue development, maintenance, and disease [57]. Using intravital imaging in murine GBM models and patient-derived cell lines, endothelial ephrin-B2 has been reported as an important regulator of vessel co-option if the ligand ephrin-B2 is also upregulated in GBM cells [58]. Moreover, the overexpression of ephrin-B2 in GBM cells was capable of transforming immortalized neural stem cells and inducing anchorage-independent growth. Importantly, genetic downregulation of ephrin-B2 impaired in vivo vessel co-option and improved survival [58].

### Olig2/Wnt7a: individual-cell versus collective-cell vessel co-option

**Fig. 3:**
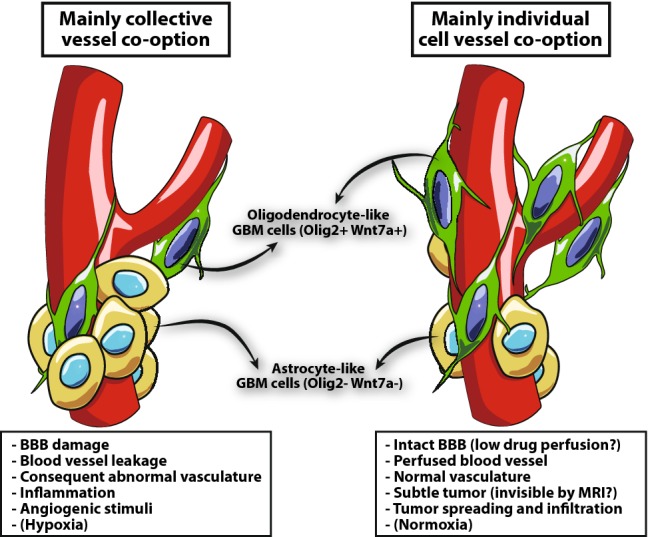
Individual versus collective-cell vessel co-option. GBMs with a prevalent collective-cell vessel co-option are probably mainly composed of astrocyte-like GBM cells. These tumors show disruption astrocyte-vascular coupling and BBB, with a consequent blood vessel leakage and abnormal vasculature (large lumen and tortuous architecture), inflammation caused by vessel leakage and an increase of angiogenic stimuli. While GBMs with more individual-cell vessel co-option are composed of a prevalence of OPC-like GBM cells. These tumors are characterized by a much more subtle infiltration of the surrounding tissue, with no inflammation and vascular leakage

Patient-derived GBM cells may co-opt blood vessels as individual cells or as a collective cluster of cells. We recently demonstrated that the Olig2-Wnt7a signaling axis clearly induces individual-cell vessel co-option, making the tumors more infiltrative [25]. These data suggest that astrocyte-like GBM cells (i.e., Olig2- and Wnt7-negative) co-opt blood vessels mainly as collective clusters of cells, while OPC-like cells (i.e., driven by Olig2 and Wnt7) spread in the surrounding tissue as individual cells in association with blood vessels [25]. Astrocyte-like GBMs, prevalently characterized by collective vessel co-option, show disruption of the astrocyte-vascular coupling and blood–brain barrier (BBB) breach, with consequent blood vessel leakage, abnormal vasculature (large lumen and tortuous architecture), and inflammation caused by vessel leakage [25, 31]. In contrast, more OPC-like GBMs with individual-cell vessel co-option are characterized by a much more subtle infiltration of the surrounding tissue, with no inflammation or vascular leakage. Thus, OPC-like tumors—or the tumor regions characterized by a prevalence of OPC-like cells—are theoretically undetectable by clinical imaging and are less inflammatory [25, 31] (Fig. 3). Importantly, Wnt7 can be targeted with inhibitors of porcupine in the endoplasmic reticulum, by reducing the secretion of multiple Wnt ligands [59]. Ex vivo and in vivo treatment with porcupine inhibitors showed a progressive reduction of cells in contact with blood vessels during Wnt inhibition. Moreover, porcupine inhibition showed chemotherapy sensitization in terms of survival of mice. Notably, vessel co-option inhibition through porcupine inhibition induced a more angiogenic phenotype, thus underlining the reciprocity between the two alternative vascular strategies [25].

## Intrinsic versus acquired resistance to anti-angiogenesis

Vessel co-option can be an intrinsic feature of specific GBM subtypes or an adaptive ability acquired as a consequence of angiogenesis inhibition, such as with bevacizumab, sunitinib, etc. [41]. Clinically, studies have shown that vessel co-option occurs in GBM patients in the peritumor regions, and is increased in patients following anti-angiogenic treatment [41, 44]. Thus, vessel co-option has been proposed as a resistance mechanism to anti-angiogenic therapy [33, 41, 60, 61].

Patients with vessel co-opting tumors are intrinsically resistant to anti-angiogenic treatment, since they are not dependent on angiogenic factors, such as VEGF, Ang-2, or the fibroblast growth factor (FGF)-2 [5, 15, 26, 62]. Further, patients with co-opting tumors have the potential to reduce the overall efficacy of anti-angiogenic therapy measured in clinical trials even if many patients in the study cohort are responders with angiogenic tumors. Currently, there are no studies describing the rates of occurrence of angiogenesis or vessel co-option-driven GBMs in patients, which limits the ability to stratify patients in the trial design. This highlights the critical need of validated biomarkers capable of stratifying GBM patients regarding the extent of vessel co-option.

A second reason for the partial failure of anti-angiogenic treatments is certainly due to high GBM cell plasticity. As demonstrated in preclinical and clinical samples, anti-angiogenesis treatments induce vessel co-option in originally angiogenic tumors [25, 44]. Unfortunately, vessel co-option inhibition has also been shown to induce pro-angiogenic factors in treated cells and tumors [25]. These data show how GBMs can switch between vessel co-option and angiogenesis to meet their metabolic needs. Therapeutically, this suggests that inhibition of both pathways may reduce the occurrence of acquired resistance mechanisms [33]. Our recent computational model suggests that sequential inhibition of vessel co-option followed by anti-angiogenesis treatment could reduce GBM growth in comparison with the simultaneous blockade [33].

## Perspective

The study of vessel co-option is an emerging field in vascular and tumor biology. It is likely that many types of tumors employ vessel co-option intrinsically or as a resistance mechanism to counter anti-angiogenic therapy. It is also conceivable that vessel co-option could influence multiple crucial features of tumors, such as hypoxia/normoxia, tumor metabolism, tumor invasion, immune suppression, and the presence of cells in specific resistance niches. While this mini-review has focused largely on vessel co-option and its relationship with angiogenesis, tumors can also recruit blood vessels by additional mechanisms: vasculogenesis, intussusception, and vascular mimicry [41, 60]. Relationships between these mechanisms and vessel co-option are virtually unknown. Our knowledge and classification of human tumors based on vessel co-option is still in its infancy and we lack histological and molecular signatures capable of stratifying GBM patients for the extent of vessel co-option. Finally, a better molecular understanding and well-characterized animal models will be needed to develop new therapeutic strategies aimed at inhibiting vessel co-option in preclinical models and in patients for combining with the approved and emerging treatments. This is even more critical as a result of the recent approvals of combinations of anti-angiogenic drugs and immune-checkpoint blockers for lung and kidney cancers [63, 64]. These trials used anti-VEGF agents that target primarily angiogenic blood vessels. However, the combination of bevacizumab (an anti-VEGF antibody) and an immune checkpoint blocking antibody has failed to improve survival of GBM patients in a randomized phase III trial. It remains unknown if and to what extent the inability of bevacizumab to target co-opted vessels in GBM played a role in this failure. Future studies need to address the role of vessel co-option in immunotherapy, a treatment that has revolutionized the therapy of more than 15 tumor types [63].
